# Tocogram characteristics of uterine rupture: a systematic review

**DOI:** 10.1007/s00404-016-4214-7

**Published:** 2016-10-08

**Authors:** Marion W. C. Vlemminx, Hinke de Lau, S. Guid Oei

**Affiliations:** 1 Department of Obstetrics and Gynecology, Máxima Medical Center, P.O. Box 7777, 5500 MB Veldhoven, The Netherlands; 2 Department of Electrical Engineering, Eindhoven University of Technology, P.O. Box 513, 5600 MB Eindhoven, The Netherlands

**Keywords:** External tocodynamometer, Hyperstimulation, Intra-uterine pressure catheter, Tocogram, Trial of labor after cesarean section, Uterine rupture

## Abstract

**Purpose:**

Timely diagnosing a uterine rupture is challenging. Based on the pathophysiology of complete uterine wall separation, changes in uterine activity are expected. The primary objective is to identify tocogram characteristics associated with uterine rupture during trial of labor after cesarean section. The secondary objective is to compare the external tocodynamometer with intrauterine pressure catheters.

**Methods:**

MEDLINE, EMBASE, and the Cochrane library were systematically searched for eligible records. Moreover, clinical guidelines were screened. Studies analyzing tocogram characteristics of uterine rupture during trial of labor after cesarean section were appraised and included by two independent reviewers. Due to heterogeneity, a meta-analysis was only feasible for uterine hyperstimulation.

**Results:**

Thirteen studies were included. Three tocogram characteristics were associated with uterine rupture. (1) Hyperstimulation was more frequently observed compared with controls during the delivery (38 versus 21 % and 58 versus 53 %), and in the last 2 h prior to birth (19 versus 4 %). Results of meta-analysis: OR 1.68 (95 % CI 0.97–2.89), *p* = 0.06. (2) Decrease of uterine activity was observed in 14–40 % and (3) an increasing baseline in 10–20 %. Five studies documented no changes in uterine activity or Montevideo units. A direct comparison between external tocodynamometer and intrauterine pressure catheters was not feasible.

**Conclusions:**

Uterine rupture can be preceded or accompanied by several types of changes in uterine contractility, including hyperstimulation, reduced number of contractions, and increased or reduced baseline of the uterine tonus. While no typical pattern has been repeatedly reported, close follow-up of uterine contractility is advised and hyperstimulation should be prevented.

## Introduction

There is a worldwide increasing incidence of cesarean sections (CS) [1, 2]. Subsequently, there will be a growing number of pregnant women with a previous uterine scar. The high success (76 %) of vaginal birth after cesarean section (VBAC) and the degree of maternal and neonatal safety have encouraged physicians and midwives to be supportive of women attempting trial of labor after the previous cesarean section (TOLAC) [3, 4]. Moreover, VBAC is advocated as a means to control the increasing rates of operative delivery [5]. Despite the excellent outcome, every physician should keep in mind the risk of a uterine rupture. Unfortunately, the incidence of uterine rupture has not declined in the last decades [6]. Women opting for TOLAC have a less than 1 % chance on a complete uterine rupture, which is associated with an estimated 10 % risk of perinatal mortality [4, 7–10].

The number of repeat CS needed to prevent one uterine rupture is very high [11]. Alternatively, intrapartum monitoring of women during TOLAC could be improved. The classical symptoms of uterine rupture are described as fetal heart rate abnormalities, the onset of severe abdominal pain persisting between contractions, scar tenderness, abnormal vaginal bleeding, hematuria, cessation of previously efficient uterine activity, loss of station of the presenting part, and maternal hypotension or shock [12]. Timely diagnosing a uterine rupture remains challenging as these symptoms can appear at a late stage or may not be present at all [3, 13–15]. In the end, the diagnosis will have to be confirmed or rejected during an emergency CS.

Clinical guidelines concerning TOLAC mainly focus on fetal heart rate abnormalities and clinical signs [12, 16]. However, based on the pathophysiology of complete uterine wall separation, changes in the uterine activity can be expected. A defect in the uterine wall reduces wall tension and can, therefore, lead to a decrease or clipping of intrauterine pressure [17]. Moreover, reduced tension can diminish contractility and influence contraction frequency and/or amplitude [18]. Therefore, uterine activity patterns, monitored by an intrauterine pressure catheter (IUPC), external tocodynamometer (TOCO), or electrohysterogram (EHG) could potentially provide warning signs of uterine rupture [19].

This systematic review aims to summarize the tocographic characteristics related to uterine rupture during TOLAC. The primary goal is to identify changes in the tocogram preceding or occurring during this emergency event. The secondary goal is to compare TOCO with IUPC.

## Materials and methods

### Sources

This systematic review was conducted according to the PRISMA guidelines. The MEDLINE, EMBASE, and the Cochrane library have been systematically searched in September 2016 using the following standardized medical subject headings (MeSH): uterine rupture, obstetric labor, trial of labor, vaginal birth after cesarean, uterine contraction, uterine monitoring, fetal monitoring, cardiotocography, tocogram, and related terms presented in the title and abstract. No limits have been used. The full electronic search strategy is available in “Appendix”. Furthermore, the references of paragraphs on intrapartum monitoring during TOLAC available in national and international guidelines (NVOG, ACOG, RCOG, and SOGC), as well as the references of the selected articles have been included. To assess eligibility of the studies, two authors (MV, HdL) independently appraised and cross checked the extracted studies. In case of disagreement, the two reviewers reconsidered the article and made the final decision.

### Study selection

**Fig. 1:**
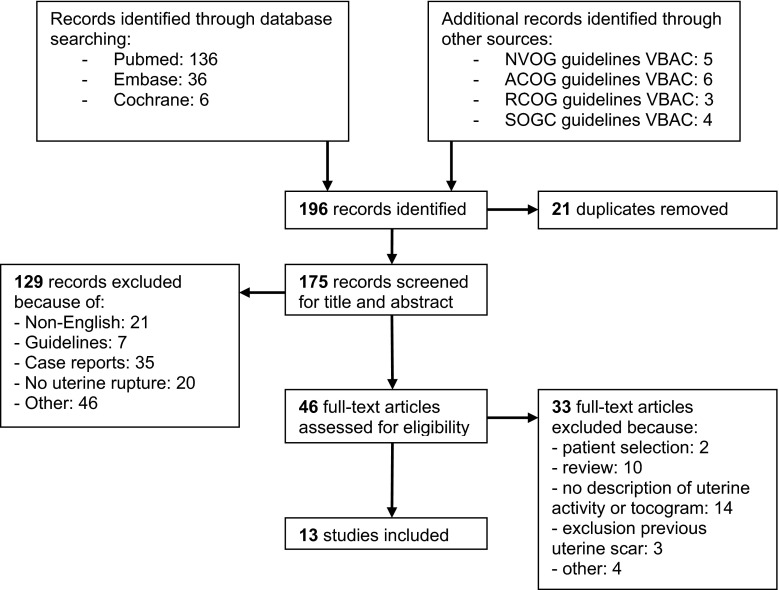
Flowchart of the reviewing process

A total of 175 articles have been systematically identified after removing duplicates. Figure 1 shows a flowchart of the search strategy and selection. We selected studies that featured an analysis of the uterine activity during TOLAC, in term pregnant women with a complete uterine rupture confirmed during CS or during postpartum complications. In each study population, there should be a minimum of five cases and at least 50 % of the women should have a previous cesarean scar. Articles not in English, case reports, reviews, and guidelines were excluded. Because of the limited amount of available evidence, the critical appraisal was restricted to study design, patient selection, and analysis of the tocogram. After reading the 46 full-text articles, the reviewers excluded two reports based on patient selection. Since a minority of the women had a previous CS, the case–control study of Sheiner et al. and the study of Chen et al. have been excluded [20, 21]. The quality of the articles was assessed using the Newcastle–Ottawa scale, which is a quality assessment tool for non-randomized studies included in meta-analysis. This scale contains eight items, which are categorized into three themes: selection (four stars), comparability (two stars), and exposure (three stars) [22]. High-quality studies achieve seven stars or more, medium quality studies between four and six stars, and poor-quality studies less than four stars.

### Statistical analysis

Data of all included studies have been extracted and subdivided into a variety of characteristics related to uterine rupture. If not provided, odds ratios and 95 % confidence interval (95 % CI) were calculated using contingency tables when possible. A meta-analysis was only considered feasible for uterine hyperstimulation during delivery due to the heterogeneity of the included studies with regard to the study design and the observed tocogram characteristics. We performed the meta-analysis in RevMan (Revision Manager 5.3 for Windows, Utrecht; Cochrane The Netherlands) and applied a random effects model. Inter-studies heterogeneity was tested using the Chi-squared test. A *p* value of <0.05 was considered statistical significant.

## Results

Thirteen studies have been included in this systematic review: one prospective cohort study, six case–control studies, and six retrospective cohort studies. The results could be categorized into five main themes: hyperstimulation, decrease in uterine activity, increased baseline, Montevideo units, or no changes in tocogram characteristics. An overview of the included studies and their results are shown in Table 1.

**Table 1:** Overview of the included studies analyzing tocogram characteristics of uterine rupture during TOLAC

Study	Design	Population	Method	Findings	Study characteristics (+/~)	Quality assessment^a^
**Hyperstimulation**						
Andersen (2016)	Case–control Denmark 1997–2008	Rupture *N* = 53, 100 % previous CS Control *N* = 43, TOLAC, 1:2, each two subsequent intended VBAC In 39,042 during TOLAC	ND	Uterine hyperstimulation was common during the last 4 h prior to delivery in both groups 58.5 % (31/53) rupture group vs. 53.5 % (23/43) controls Sub analyses of first stage, second stage, induction, and augmentation of labor: no significant differences.	+FIGO guidelines: tachysystole >5/10 min +19 blinded experts; 3 for each tracing +Terminal bradycardia was removed ~28 % no evaluation tocogram last hour prior to delivery due to poor-quality tracing	8 (4/1/3)
Craver Pryor (2007)	Case–control USA 1995–2000	Rupture *N* = 26, 100 % previous CS Control *N* = 52, successful VBAC, 1:2, each VBAC before/after rupture In 1,896 during TOLAC	ND	More hyperstimulation <2 h prior to delivery 19.2 vs. 3.8 %, OR 5.9 (95 %CI 1.2–28.6), *p* < 0.05 No significant differences for <4 and 2–4 h	+Hyperstimulation >5/10 min +Two blinded independent reviewers ~Uterine rupture; significant longer duration and higher maximum dose of oxytocin	7 (4/1/2)
Goetzl (2001)	Case–control USA 1984–1996	Rupture *N* = 24, 100 % previous CS Control *N* = 96, TOLAC Matching; 1:4, by birth weight, year of delivery, induced/augmented In 1650 primiparas during TOLAC	ND	Cases were more likely to have an episode of uterine hyperstimulation Overall: 37.5 vs. 20.8 %, *p* = 0.05 Induction: 38.5 vs. 30.8 %, *p* = 0.42 Augmentation: 36.4 vs. 9.1 %, *p* = 0.05	+Hyperstimulation >5/10 min, that resulted in a reduction of oxytocin administration. +Analysis of oxytocin use: no difference ~Investigators unknown; How many? Blinded? ~Physician-based diagnosis in the medical file; No CTG monitoring strips	7 (4/2/1)

Study	Design	Population	Method	Findings	Study characteristics	
**Decrease of uterine activity**						
Zwart (2009)	Prospective cohort Netherlands 2004–2006	Rupture *N* = 210, 87 % previous CS In 358,874 deliveries	ND	13.6 % (25/184) acute absence of contractions	+Large nationwide prospective study ~No control cohort ~Definition acute absence?	4 (3/0/1)
Ridgeway (2004)	Case–control USA/Sweden 1983–2001	Rupture *N* = 36, 100 % previous CS Control *N* = 100, successful VBAC Matching; 1:3, by GA and institution In 45,113 deliveries	ND	Loss of uterine tone during 4 h before second stage and during the second stage: first stage (*N* = 36); 2.8 % (*N* = 1) vs. 0 % *p* = 0.27 second stage (*N* = 14); 0 vs. 0 % *p* = not applicable	+3 Independent and blinded examiners ~Definition loss of uterine tone?	8 (4/2/2)
Phelan (1998)	Case control USA 1994	Rupture *N* = 18, 94 % previous CS Control (1) *N* = 35 successful VBAC Control (2) *N* = 33 SVD Controls delivered in Nov–Dec 1994; all VBAC and every 20th SVD	Most TOCO	Study cases had significant fewer contractions per hour compared with both control groups: 15.8 rupture versus 19.7 VBAC, *p* = 0.03 15.8 rupture versus 19.4 SVD, *p* = 0.09	+ From active phase of labor; 4 cm dilation ~ Selection method: severe neonatal brain injury ~ One investigator, not blinded	4 (2/1/1)
Arulkumaran (1992)	Retrospective cohort Singapore 1985–1990	Rupture *N* = 9, 100 % previous CS In 722 women during TOLAC	IUPC: 56 %	33.3 % (3/9) showed decrease in uterine activity, which were all registered with IUPC	~4/9 incomplete rupture = intact serosa ~Definition of decrease?	5 (3/0/2)
Beckley (1991)	Retrospective cohort England 1982–1988	Rupture *N* = 10, 100 % previous CS In 1740 women during TOLAC	IUPC 100 %	40.0 % (4/10) show marked fall in uterine activity; ‘clipping’ off of the pressure peaks	+Tocogram tracings available in article ~Definition marked fall uterine activity?	5 (3/0/2)

Study	Design	Population	Method	Findings	Study characteristics	
**Increasing baseline**						
Zwart (2009)	Prospective cohort Netherlands 2004–2006	Rupture *N* = 210, 87 % previous CS In 358,874 deliveries	ND	20.2 % (38/188) hypertonia	+Large nationwide prospective study ~Definition of hypertonia?	4 (3/0/1)
Rodriguez (1989)	Retrospective cohort USA 1979–1988	Rupture *N* = 76, 79 % previous CS In 138,853 deliveries	IUPC: 51 %	Increase of baseline uterine tone in 10.2 % (4/39) using an IUPC. Decrease of uterine activity was not observed with IUPC (*n* = 39)	~Definition increasing baseline?	5 (3/0/2)

Study	Design	Population	Method	Findings	Study characteristics	
**Montevideo units**						
Maggio (2014)	Case–control USA 2007–2010	Rupture *N* = 9, 100 % previous CS Control 1, *N* = 48, successful VBAC Control 2, *N* = 35, failed TOLAC In 986 women attempting TOLAC	IUPC 100 %	The MVU in 120 min prior to uterine rupture, CS or complete dilation in VBAC were similar among all groups: medians ranged, respectively, 130–195, 145–190, and 140–175	+ 120 min of labor with IUPC +/~ Local protocol: IUPC in case of oxytocin augmentation during TOLAC and aim MVU’s ≤ 200 ~ Two investigators were not blinded	6 (4/1/1)
Buhimschi (2005)	Retrospective cohort USA 1991–2000	Rupture *N* = 26, 100 % previous CS In 96,590 deliveries	IUPC 100 %	During at least 1 h prior to uterine rupture, the average MVU/10 min [range] was 205 [160–300] oxytocin group and 247 [140–380] prostaglandin/oxytocin group	+Two independent investigators ~15 analysis based on notes or records; no CTG ~No comparison with controls	5 (3/0/2)

Study	Design	Population	Method	Findings	Study characteristics	
**No changes in uterine activity**						
Menihan (1998)	Retrospective cohort USA 1990–1995	Rupture* N* = 11, 100 % previous CS In 3353 women during TOLAC	IUPC: 36 %	All normal uterine activity before onset bradycardia. Decrease/cessation uterine tone was not observed	~Definition decrease or cessation?	5 (3/0/2)
Phelan (1998)	Case control USA 1994	Rupture *N* = 18, 94 % previous CS Control (1) *N* = 35 successful VBAC Control (2) *N* = 33 SVD Controls delivered in Nov–Dec 1994; all VBAC and every 20th SVD	Most TOCO	Hyperstimulation: 61 % rupture, 74 % VBAC, 64 % SVD Tetanic episodes: 67 % rupture, 54 % VBAC, 85 % SVD. and no significant differences between groups	+Hyperstimulation >5/10 min +Tetanic episode = contraction >90 s ~Selection method: severe neonatal brain injury ~One investigator	4 (2/1/1)
Leung (1993)	Retrospective cohort USA 1983–1992	Rupture *N* = 86, 100 % previous CS In 11,179 during TOLAC	ND	Decrease of uterine tone or cessation of uterine activity has not been observed	+Complete, partial or no fetal extrusion ~Definition decrease or cessation?	4 (3/0/1)
Rodriguez (1989)	Retrospective cohort USA 1979–1988	Rupture *N* = 76, 79 % previous CS In 138,853 deliveries	IUPC: 51 %	Decrease of uterine activity was not observed with IUPC (*n* = 39)	~Definition decrease?	5 (3/0/2)

The studies are categorized according to five themes: hyperstimulation, decrease of uterine activity, increasing baseline, Montevideo units, and no changes in uterine activity

Method = uterine monitoring technique

^a^Quality assessment according to Newcastle–Ottawa quality assessment scale for case–control and cohort studies (selection/comparability/outcome, maximum score 9)

+, Positive study characteristic; ~, Negative study characteristic

### Hyperstimulation

The frequency of contractions prior to uterine rupture has been examined in three case–control studies. In the study by Goetzl et al., uterine rupture was more often preceded by an episode of hyperstimulation (defined as >5 contractions per 10 min, that resulted in reduced administration of oxytocin) compared with controls: 37.5 and 20.8 %, *p* = 0.05, which is on the margin of significance [23]. Odds ratios were not provided. Craver Pryor et al. studied hyperstimulation at more than 4, 2–4, and less than 2 h prior to delivery. Hyperstimulation (defined as >5 contractions per 10 min) was more common during the 2 h prior to birth: 19.2 and 3.8 %, *p* < 0.05 (OR 5.9, CI 1.2–28.6) [24]. In contrast, a more recent study of Andersen et al. showed no significant difference in uterine hyperstimulation (>5 contractions per 10 min) during labor: 58.5 % in the rupture group versus 53.5 % in controls, *p* = 0.74 [25]. Subanalyses in the first/second stage and induced/augmented labor also showed no significant differences in their study. All three case–control studies did not report how the uterine activity patterns were monitored.

### Meta-analysis of hyperstimulation

**Fig. 2:**
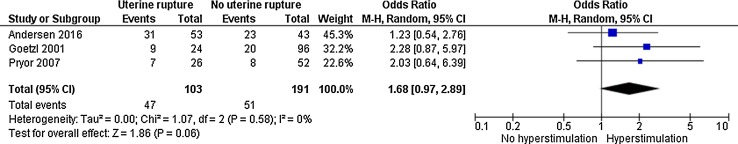
Review: tocogram characteristics related to uterine rupture. Comparison: hyperstimulation and no hyperstimulation during trial of labor after the previous cesarean section. Outcome: risk of uterine rupture

A meta-analysis was performed based on the three above-mentioned case–control studies evaluating uterine hyperstimulation during TOLAC in relation to the risk of uterine rupture (see Fig. 2). Uterine hyperstimulation during TOLAC showed a trend in relation to the risk of uterine rupture: OR 1.68 (95 % CI 0.97–2.89), *p* = 0.06. The Chi-squared test for inter-study heterogeneity was non-significant (*p* = 0.58).

### Decrease in uterine activity

In a large nationwide Dutch prospective cohort study of Zwart et al., acute absence of contractions was reported in 13.6 % (25/184) of the cases of uterine rupture [10]. They did not describe the applied uterine monitoring techniques. A smaller case–control study of Ridgeway et al. focused on fetal heart rate characteristics of patients with uterine rupture compared with successful VBAC. They described loss of uterine tone during the first stage in a single case (1/36) [14]. Two small retrospective studies of Arulkumaran et al. and Beckley et al. found a decrease of the uterine contraction amplitude in, respectively, 33.3 % (3/9) and 40.0 % (4/10) of the uterine ruptures, which were all monitored by IUPC [26, 27]. Finally, Phelan et al. observed a significantly (*p* = 0.03) lower amount of contractions per hour in ruptures (15.8/h) compared with VBAC (19.7/h), monitored from the onset of active labor defined as cervical dilation of 4 cm [28]. This difference was not significantly different when comparing only oxytocin recipients; 16.5 contractions per hour in the rupture group, and 18.1 contractions per hour in VBAC. Most of their cases had external fetal monitoring.

### Increasing baseline

Zwart et al. observed hypertonia in 20 % (38/188) of the uterine ruptures in their large nationwide prospective study [10]. They did not describe their definition of hypertonia or which uterine monitoring technique (i.e., TOCO or IUPC) was applied. The retrospective study of Rodriguez et al. detected an increased baseline uterine pressure in 10 % (*n* = 4) of the uterine rupture cases (*n* = 39) which were monitored with an IUPC [29].

### Montevideo units

Montevideo units (MVU) can only be calculated in the presence of an IUPC. In the case–control study of Maggio et al., cases of uterine rupture have been compared with successful VBAC and failed TOLAC [35]. They found no association between MVU and uterine rupture in pregnant women undergoing TOLAC. Over time, MVU showed a continued increase during the last 2 h prior to birth in the successful VBAC group (*p* < 0.01) and lack of such increase in the rupture group (*p* = 0.26). However, when only using the first stage of labor, there was no difference in MVU over time between uterine rupture versus VBAC (*p* = 0.22) and uterine rupture versus failed TOLAC (*p* = 0.87).

Buhimschi et al. retrospectively investigated the uterine rupture localization associated with prostaglandins treatment. Therefore, they compared uterine ruptures during TOLAC in women who received prostaglandins + oxytocin versus uterine ruptures in women with oxytocin alone [30]. The average amount of MVU was 205 (range 160–300) per 10 min in the oxytocin only group compared with 247 (range 140–380) per 10 min in the prostaglandin/oxytocin group, during at least 1 h prior to rupture [30]. These results were not compared with controls.

### No change in uterine activity

Uterine activity patterns of uterine ruptures resulting in permanent severe brain injury have been examined by Phelan et al. [28]. No significant difference in the occurrence of hyperstimulation or tetanic episodes was found. A retrospective study of Menihan et al. focused on both features of fetal heart rates and uterine activity patterns in 11 cases of uterine rupture with 36 % (4/11) IUPC monitoring; no change in uterine activity was found [31]. Leung et al. analyzed uterine activity amongst numerous other features in 86 cases of uterine rupture during TOLAC and observed no decrease of uterine tone or cessation of contractions. Their tocographic method was not described [32]. Finally, Rodriguez et al. also observed no decrease in 39 cases monitored with IUPC [29].

## Discussion

In this systematic review of the literature, several changes in uterine activity have been identified to be associated with uterine rupture: hyperstimulation, decrease in uterine activity, and an increased or reduced baseline tonus. Of these tocogram characteristics, only hyperstimulation could be evaluated in a meta-analysis: showing an increased risk of uterine rupture in case of hyperstimulation, on the margin of significance (*p* = 0.06). Furthermore, in a large prospective study, hypertonia was reported in 20 % of the cases and acute absence of contractions in 14 % [10].

We are aware that the majority of the included studies are of retrospective design (12 out of 13). Since uterine rupture is a relatively rare event, retrospective study designs are commonly used. However, this carries the risk of selection bias. For example, Phelan et al. identified their cases within the National Registry of Brain Injured Babies, including only those uterine ruptures resulting in severe perinatal morbidity or ‘silent’ uterine ruptures potentially leading to selection bias [28]. The size of the retrospective study populations also showed a strong variation, from 9 up to 86 cases of uterine rupture. In addition, the two large retrospective studies showed dissimilar results compared with the single prospective study: Leung et al. (*n* = 86) and Rodriguez et al. (*n* = 39) observed no decrease of uterine activity [29, 32], while Zwart et al. revealed acute absence of contractions in 14 % of uterine rupture cases (25 out of 184) in their prospective study [10]. Furthermore, our systematic search identified multiple large studies regarding uterine ruptures in which information on the tocogram was not provided, which could entail publication bias. For example, Al-Zirqi et al. (*n* = 94) and Kwee et al. (*n* = 98) identified a total of 192 uterine ruptures, yet both studies did not analyze uterine activity patterns [8, 33]. And we excluded the study of Kayani et al., because there was no uterine activity evaluation, while they do report that ‘the intrauterine pressure catheters recording have contributed to the diagnosis of uterine rupture’ [34].

In this systematic review, we are interested in tocogram characteristics of complete uterine rupture during TOLAC. Uterine rupture concerns a challenging diagnosis. This is reflected in the diverse definitions of uterine rupture in the included studies. A complete uterine rupture, defined as disruption of all the layers of the uterine wall resulting in direct communication between the uterine cavity and peritoneal cavity, might result in different symptoms than dehiscence of the uterine scar, in which case the serosa is still intact leading to minimal intraabdominal bleeding and often few or no symptoms. Several studies identified their cases based on the International Classification of Diseases (ICD-9) coding for uterine rupture during labor, which does not discriminate between a complete rupture and dehiscence [14, 24, 31]. Furthermore, we are aware that not all cases of the included studies concerned women with a previous uterine scar (79–100 %). Finally, uterine activity parameters have not been clarified in some studies. For example, Zwart et al. described hypertonia in 20 % of the uterine rupture cases, but did not define hypertonia [10], whereas studies examining a decrease in uterine activity did not provide a percentage in decrease. Therefore, our systematic review might consist of a mix of both complete and incomplete uterine ruptures, scarred and unscarred uteri, and uterine activity characteristics might be indistinct.

Continuous electronic fetal monitoring is recommended during TOLAC, whilst there is no consensus about the method for monitoring contractions [12, 16]. International guidelines concerning TOLAC do not recommend routine use of IUPC’s as they do not assist in the diagnosis of uterine rupture [12, 16]. Yet, compared with TOCO, an IUPC has the advantage of providing quantitative measurement of uterine resting tone as well as the intensity and MVU of contractions, possibly contributing to the diagnosis of a uterine rupture. Unfortunately, in this systematic review, half of the studies do not document their tocographic method, impeding the comparison of the two modalities used for monitoring uterine contractions. Two features of the tocogram, however, a decrease in contraction amplitude and increasing baseline pressure, are only observed using an IUPC. Rodriguez et al. noticed an increase of baseline uterine tone in 4 out of 39 women monitored IUPC, while not visible in the 29 women monitored with TOCO [29]. This might indicate that an IUPC is needed to observe these subtle changes in the tocogram. The use of IUPC during TOLAC is not supported by Maggio et al. who found no differences in MVU between uterine ruptures and VBAC [35]. Devoe et al. also revealed no change in uterine tone and peak uterine pressure 2.5 min after uterine incision for CS [36]. Possibly, the observed changes can also be influenced by localization of the catheter [26]. The results of this review do not provide solid evidence for the standard use of an IUPC. Nevertheless, this does not negate the need for adequate uterine monitoring during TOLAC.

The observed association of hyperstimulation and uterine rupture has no trivial relation. The relationship could be causal in nature in the sense that hyperstimulation by oxytocin administration leads to increased stress on the uterine scar and eventually failure. Alternatively, failure of the scar could cause an increase in contraction frequency due to intraabdominal blood causing excitation of the myometrium, in this way preceding a complete rupture. However, based on the physiology of uterine contractions, a decrease rather than an increase in contraction frequency caused by a loss of wall tension is to be expected [18, 37]. The combination is also conceivable and could explain why both changes in contraction frequency were observed: hyperstimulation causing rupture of the scar and then leading to a cessation of uterine contractions. It is remarkable that the only study of ruptures with severe neonatal brain injury showed significant less contractions in the uterine rupture group, which might indicate that the disastrous event has already occurred [28]. Finally, it could also be a confounding factor, associated with, for instance, prolonged deliveries, abnormal fetal presentation, or macrosomia. The information available does not permit further analysis of this relationship.

In literature, fetal heart rate abnormality is the most common sign associated with uterine rupture, which has been reported in up to 70 % of the cases of uterine rupture [16]. Andersen et al. even revealed that none of the uterine rupture cases had a completely normal CTG according to the International Federation of Gynecology and Obstetrics (FIGO) guidelines [25]. Only a great number of severe variable decelerations, fetal bradycardia, or preterminal CTG were significant pathologic fetal heart pattern to differentiate uterine rupture from successful VBAC [14, 25]. We calculated the positive predictive values of several fetal heart rate and uterine activity patterns in the study of Ridgeway et al., based on a contingency table and corrected for an estimated uterine rupture prevalence of 1.0 %. For example, the estimated positive predictive value for bradycardia in the second stage was 8.3 %. In addition, the positive predictive value of mild–moderate and severe variable decelerations in the first stage was, respectively, 1.2 and 4.0 % [14]. Andersen et al. showed comparable low diagnostic values of fetal heart rate characteristics [25]. This compares to the predictive value of uterine hyperstimulation of 4.8 % less than 2 h prior to delivery evaluated in the study of Pryor et al. [24]. Hence, a pathological CTG cannot be considered as a strong predictor of uterine rupture [25]. Physician decision-making should, therefore, be based on monitoring clinical signs, fetal heart rate patterns, and uterine activity during TOLAC [25].

International guidelines report a two to threefold increased risk of uterine rupture during induction and augmentation of labor [12, 25, 38]. This could be related to the increased risk of uterine hyperstimulation due to oxytocin usage. In the study of Craver Pryor et al., uterine rupture cases experienced a significant longer duration of oxytocin and maximum dose of oxytocin compared with controls [24]. However, no significant differences in oxytocin were reported by Goetzl et al. [23]. These somewhat contradictory results support to at least closely monitor the use of oxytocin to prevent hyperstimulation. Therefore, special attention should be paid to monitor the contraction frequency and to correct the frequency pattern as necessary. Unfortunately, substandard care during TOLAC is a common problem. For example, a proper assessment of the uterine activity could not be made in 28 % of the cases in last hour prior to uterine rupture in the study of Andersen et al. [25]. Moreover, the current guidelines do not recommend a strict contraction frequency. Based on our results, we would advise to aim for 3–5 contractions per 10 min. More than 5 contractions per 10 min should be corrected with oxytocin reduction or tocolytic drugs. And if no adequate tocogram can be obtained with TOCO, alternative tocographic techniques like an IUPC or an EHG-based method should be considered to guarantee adequate uterine monitoring and to prevent hyperstimulation [39, 40].

## Conclusion

Uterine rupture can be preceded or accompanied by several types of changes in uterine contractility, including hyperstimulation, reduced number of contractions, increased or reduced baseline tonus. While no typical pattern has been repeatedly reported, we advise close follow-up of uterine contractility for the early detection of atypical changes, and to prevent uterine hyperstimulation.

## Appendix: Systematic literature searches on September 20th 2016

Uterine rupture[Mesh] OR uterine rupture[tiab] OR scar rupture[tiab] = #5336

AND

Labor, Obstetric[Mesh] OR Labor[tiab] OR Trial of labor[Mesh] OR Trial of labor[tiab] OR Trial of labor after cesarean [tiab] OR Vaginal birth after cesarean[Mesh] OR VBAC[tiab] OR uterine scar[tiab] = #91729

AND

Uterine contraction[Mesh] OR uterine contraction[tiab] OR contraction*[tiab] OR hyperstimulation[tiab] OR uterine monitoring[Mesh] OR uterine monitoring[tiab] OR uterine activity[tiab] OR uterine tone[tiab] OR uterine patterns[tiab] OR Cardiotocography[Mesh] OR cardiotocography[tiab] OR Fetal monitoring[Mesh] OR fetal monitoring [tiab] OR tocogram[tiab] OR external tocodynamometry [tiab] OR intrauterine pressure catheter[tiab] OR intrauterine pressure[tiab] = #148425

-># 136 records

*Search EMBASE* (uterine rupture OR scar rupture) AND (trail of labor OR obstetric labor OR vaginal birth after cesarean OR VBAC OR uterine scar) AND (uterine contraction OR contraction* OR uterine activity OR cardiotocography OR fetal monitoring OR tocogram OR external tocodynamometry OR intrauterine pressure) -># 36 records

*Search Cochrane* Uterine rupture AND trial of labor AND cardiotocography -># 6 records

## Abbreviations

CS: Cesarean section
EHG: Electrohysterography
GA: Gestational age
IUPC: Intrauterine pressure catheter
MVU: Montevideo units
SVD: Spontaneous vaginal delivery
TOCO: External tocodynamometer
TOLAC: Trial of labor after previous cesarean section
VBAC: Vaginal birth after cesarean section
